# The Relationship of Shopping-Related Decisions with Materialistic Values Endorsement, Compulsive Buying-Shopping Disorder Symptoms and Everyday Moral Decision Making

**DOI:** 10.3390/ijerph19074376

**Published:** 2022-04-05

**Authors:** Astrid Müller, Ekaterini Georgiadou, Annika Birlin, Nora M. Laskowski, Susana Jiménez-Murcia, Fernando Fernández-Aranda, Thomas Hillemacher, Martina de Zwaan, Matthias Brand, Sabine Steins-Loeber

**Affiliations:** 1 Department of Psychosomatic Medicine and Psychotherapy, Hannover Medical School, 30625 Hannover, Germany; birlin.annika@mh-hannover.de (A.B.); laskowski.nora@mh-hannover.de (N.M.L.); dezwaan.martina@mh-hannover.de (M.d.Z.); 2 Department of Psychiatry and Psychotherapy, Paracelsus Medical University Nuremberg, 90419 Nuremberg, Germany; ekaterini.georgiadou@klinikum-nuernberg.de (E.G.); thomas.hillemacher@klinikum-nuernberg.de (T.H.); 3 Institute of Psychology, Technische Universität Braunschweig, 38106 Braunschweig, Germany; 4 University Clinic for Psychosomatic Medicine and Psychotherapy, Medical Faculty, Campus East-Westphalia, Ruhr-University Bochum, 32312 Luebbecke, Germany; 5 Ciber Fisiopatología Obesidad y Nutrición (CIBERObn), Instituto de Salud Carlos III, 28029 Madrid, Spain; sjimenez@bellvitgehospital.cat (S.J.-M.); ffernandez@bellvitgehospital.cat (F.F.-A.); 6 Psychoneurobiology of Eating and Addictive Behaviors Group, Neurosciences Programme, Bellvitge Biomedical Research Institute (IDIBELL), 08908 Barcelona, Spain; 7 Department of Psychiatry, University Hospital of Bellvitge, L’Hospitalet de Llobregat, 08907 Barcelona, Spain; 8 Psychiatry and Mental Health Group, Neuroscience Program, Institut d’Investigació Biomèdica de Bellvitge-IDIBELL, L’Hospitalet de Llobregat, 08907 Barcelona, Spain; 9 General Psychology, Cognition and Center for Behavioral Addiction Research (CeBAR), University of Duisburg-Essen, 47057 Duisburg, Germany; matthias.brand@uni-due.de; 10 Erwin L. Hahn Institute for Magnetic Resonance Imaging, 45141 Essen, Germany; 11 Department of Clinical Psychology and Psychotherapy, Otto Friedrich University of Bamberg, 96047 Bamberg, Germany; sabine.steins-loeber@uni-bamberg.de

**Keywords:** compulsive buying-shopping disorder, shopping decisions, materialism, Pathological Buying Screener, moral decision-making

## Abstract

Background: Compulsive buying-shopping disorder (CBSD) is associated with high materialistic values endorsement and excessive purchasing of consumer goods. A subgroup of individuals with CBSD engage in socially unacceptable behaviors to continue shopping despite negative consequences. This investigation aimed at exploring possible links between ego-oriented shopping-related decisions, materialism, symptoms of CBSD and close-to-everyday moral decision making. Methods: In study 1, patients with CBSD were interviewed to develop a list of conflict situations, capturing typical shopping-related dilemmas. In study 2, the shopping-related dilemmas from study 1, standardized close-to-everyday moral dilemmas, the Material Values Scale and Pathological Buying Screener were administered to a web-based convenience sample (*n* = 274). Results: The main effects of a moderated hierarchical regression analysis revealed an association of more ego-oriented shopping-related decisions with both higher materialistic values endorsement and more CBSD symptoms, but not with everyday moral decision-making. However, a more egoistic everyday moral decision making style moderated the effect of CBSD symptoms on ego-oriented shopping related decisions. Conclusions: The findings indicate that a more egoistic everyday moral decision making style is not directly linked to domain-specific shopping-related decision making but strengthens the link between symptoms of CBSD and ego-oriented shopping-related decisions.

## 1. Introduction

Shopping is a popular legitimate pastime that can become dysfunctional if it represents the main source of gratification or primary response to negative mood states. The problematic consumption behavior is associated with diminished control over purchasing and the behavior is continued or even escalated despite negative consequences (e.g., financial problems, familial discord, clinical distress) [1,2]. Dysfunctional, excessive buying/shopping seems to be prevalent across different cultures, with about 5% of adults being affected [3]. While “compulsive buying-shopping disorder (CBSD)” is currently mentioned as an example for “other specified impulse control disorders” in the ICD-11 coding tool [4], it has been argued that it may better fit the category “other specified disorders due to addictive behaviors” based on similarities with substance-related addictions and gambling and gaming disorders (e.g., reward seeking, cue-induced craving responses, diminished top-down control, disadvantageous decision making) [5,6,7].

As CBSD progresses, financial problems arise. These can lead to indebtedness and bankruptcy due to the insensitivity to long-term consequences of excessive purchasing [8,9,10,11]. At the same time, the inappropriate shopping behavior becomes more and more habitual, which is in line with theoretical models of addictions that propose an increase in compulsive or seemingly automatic behaviors in later stages of the addiction process [12,13]. Individuals with CBSD are often in desperate financial situations and increasingly confronted with conflicting shopping-related scenarios in which they have to decide between a more ego-oriented (“to buy”) and a more norm-oriented (“not to buy”) option. In the long run, many individuals with CBSD engage in socially unacceptable behaviors to fund their consumption habit [8,14,15]. According to case vignettes, the quality of these behaviors can vary greatly, ranging from dishonesty to deception, fraud, shoplifting, embezzlement to income-generating crime [10,11,15]. Although the norm-breaking behaviors are a major problem for a subgroup of individuals with CBSD and their families, they are still poorly understood. By analogy with gambling disorder [16,17], the rule-breaking activities of many individuals with CBSD could be seen as a product of the addictive behavior (i.e., addictive shopping) itself or as a qualifier of CBSD severity.

With respect to rule-breaking and socially unacceptable shopping behaviors, the following aspects are important to keep in mind. First, such behaviors are prevalent in many, but not all individuals with CBSD. Second, they may occur not only in consumers who suffer from CBSD, but also in those without CBSD [18]. Therefore, additional consumer facets should be considered to better understand the phenomenon, e.g., values that focus on materialistic needs and desires.

According to Belk [19], materialism can be viewed as the combined personality traits of envy, non-generosity, and possessiveness. Richins [20]; p. 210, described materialism as a set of values referring to “the importance ascribed to the ownership and acquisition of material goods in achieving major life goals” such as happiness, satisfaction and success. Research suggests a robust link between materialistic values endorsement and CBSD [21,22,23,24,25,26,27,28]. Some authors consider the combination of identity problems and materialism as an antecedent of CBSD [22,23,29,30]. Identity problems can make consumers vulnerable for the belief that the possession of material goods will help them to gain identity, happiness, and success, which may increase their risk to develop CBSD [22]. Based on empirical work, Dittmar [23] proposed a two-factor model that conceptualizes CBSD as identity-seeking consumer behavior that is driven jointly by materialistic values and self-discrepancy. In line with that model, Claes et al. [22] found that materialistic values endorsement mediated the association between identity confusion and CBSD in a Flemish community sample.

The priority individuals place on materialism is associated with a strong drive for possessions, ego-oriented shopping motivations, high purchasing involvement and accumulation of debt [31]. Individuals with high materialistic values endorsement may be less sensitive to activities that are harmful to others [19]. From this perspective, high materialistic values endorsement lowers ethical standards [32], which may result in norm-breaking shopping-related behaviors to gain possessions [30]. It seems plausible that high materialistic values endorsement is related to more norm-breaking and socially unacceptable shopping-related decisions, in combination or independently from CBSD.

Considering that many but not all individuals with CBSD and some but not all consumers with high material value orientation tend to make shopping-related decisions that deviate from the norm, this raises the question of whether these associations are moderated by consumers’ moral decision making style. Moral decision making refers to decisions that are subject to generally accepted, normative behaviors. It is usually investigated using moral dilemma vignettes in which there is a forced choice between two contrary courses of action by rival moral reasons [33]. While altered moral decision making has been linked to antisocial, rule-breaking behaviors and psychopathy [34,35,36], its relationship with materialism and CBSD is understudied. To our knowledge, no study on the possible connection between CBSD and moral decision making has been published so far. Research on the impact of moral decision making on consumer behavior has focused primarily on ethical consumerism, which refers to consumer behavior that is not harmful to the environment or society (consumption of ethically produced goods, green buying, etc.) [37,38].

Taken together, there is a lack of research addressing disadvantageous, self-centered, norm-breaking, socially unacceptable shopping responses in individuals with CBSD that result in negative consequences in the areas of personal finance, relationships, post-purchase distress and legal problems. The present study was conducted to shed more light on this malfeasance and to improve the understanding of the relationship of such norm violations with CBSD, materialism and moral decision making. The empirical work presented here consists of a qualitative study (study 1) and a quantitative web-based survey (study 2). Thereinafter, we will use the term “ego-oriented shopping-related decisions” to label norm-breaking shopping-related responses.

### 1.1. Aims

**Figure 1:**
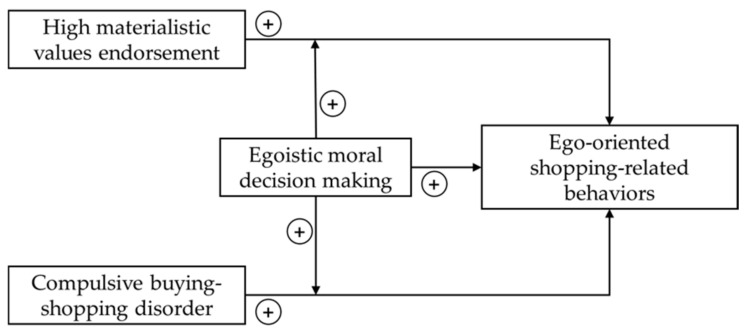
Proposed relationships of ego-oriented shopping-related behaviors with materialism, compulsive buying-shopping disorder and everyday moral decision making.

The main objective of the study was to explore the relationship of ego-oriented shopping-related decisions with CBSD, materialism and everyday moral decision making and to clarify whether an egoistic moral decision-making style moderates the relationship between materialism/CBSD and ego-oriented shopping-related decisions (see Figure 1). Given the paucity of paradigms to assess ego-oriented shopping-related decision making in relation to CBSD, patients with CBSD were interviewed to develop a list of exemplary scenarios representing a decisional conflict, capturing typical shopping-related dilemmas in study 1. In study 2, the shopping-related scenarios from study 1, standardized close-to-everyday moral dilemmas, and measures for materialistic values endorsement and CBSD were administered to a web-based convenience sample.

### 1.2. Hypotheses

Based on the above considerations, the following hypotheses were drawn:

1. Higher material values endorsement is related to more ego-oriented shopping-related decisions.
2. More symptoms of CBSD are related to more ego-oriented shopping-related decisions.
3. A more egoistic everyday moral decision making style is related to more ego-oriented shopping-related decisions.
4. A more egoistic everyday moral decision making style strengthens the association between materialistic values endorsement/symptoms of CBSD and ego-oriented shopping-related decisions.

Furthermore, we expected to replicate past findings [21,22,23,24,25,26,27,28] regarding the positive correlation between materialistic values endorsement and CBSD symptoms.

## 2. Study 1: Development of Shopping-Related Dilemmas

### 2.1. Material and Methods

#### 2.1.1. Procedure

From December 2018 to January 2019, individual open-ended interviews with CBSD patients were carried out to generate shopping-related dilemmas. Participants were asked to retrospectively describe three shopping-related conflict situations that had occurred in their daily life. For each conflict situation, they were asked to share their decision and assess whether it was rather ego-oriented or norm-oriented. The interviews lasted about 10 to 15 min, were audio recorded and transcribed verbatim. After interviews were completed, two independent researchers (A.B., N.M.L.) read the narratives to identify themes and patterns of typical shopping-related conflict situations. Following the concept of data saturation, further interviews were conducted until no new relevant and important information emerged [39]. Inspired by forced-choice moral dilemmas [40,41], an initial list of scenarios with a more “ego-oriented” and more “norm-oriented” response alternative (answered with “yes” or “no”) was created. This list was then carefully evaluated by two other researchers (A.M., E.G.) with respect to wording, clarity, ecological relevance and potential duplicates. Consensus on final themes was reached in a third round during a consensus meeting of the study 1 team (E.G., A.B., N.L.M., A.M.).

#### 2.1.2. Instruments

Symptom severity of CBSD was assessed by means of the original German version of the Pathological Buying Screener (PBS) [42]. The questionnaire consists of 13 items (e.g., “How often does it occur that you can’t stop thinking about buying?” or “… that you suffer distress from your buying habits?”) answered on a scale ranging from 1 (=never) to 5 (=very frequently). PBS total scores, which are higher (range 13 to 65), are related to higher severity of CBSD. At the recommended cut-off point of ≥29 the PBS has a sensitivity of 98% and a specificity of 94.7% [43].

#### 2.1.3. Participants with CBSD

All individuals who participated in study 1 were outpatients seeking treatment for CBSD at the Department of Psychiatry and Psychotherapy, Paracelsus Medical University Nuremberg, or the Department of Psychosomatic Medicine of Hannover Medical School. Both departments offer treatment for adults only. Inclusion criteria were age 18 years or older, the presence of CBSD based on routine clinical assessments and scores above the threshold for CBSD on the PBS [42,43]. Exclusion criteria were insufficient German language skills, psychosis, any developmental disorder, and cognitive impairments (based on clinical assessments). None of the eligible patients met any of the exclusion criteria. Participation in the study was completely voluntary, and all participants gave written informed consent to participate in study 1.

### 2.2. Results

Interviews were conducted with 14 outpatients with CBSD (13 women, 1 man) who met the inclusion criteria. Their age range was between 21 and 63 years (*Mdn* = 46). All patients answered the PBS before starting treatment and scored above the PBS threshold for CBSD (*Mdn* = 55, range 43 to 65). The self-reported median duration of CBSD in the current clinical sample was 16 years (range of 3 to 35 years).

The inspection of the verbatim transcripts revealed an initial list of 30 shopping-related conflict situations. Vignettes with substantial overlap were collapsed, resulting in a final list of 22 shopping-related dilemmas presented in Table 1.

## 3. Study 2: Online Survey

### 3.1. Material and Methods

#### 3.1.1. Procedure

Data were collected from June to September 2019 within an online survey using SoSci Survey Version 2.5.00-i (SoSci Survey GmbH, Munich, Germany). The survey was promoted via social media channels (e.g., Facebook). Customers with an age of 18 years and above were invited to participate in a “Survey on buying-related decisions”. The cover page of the survey explained the aims of the study and its academic, confidential and anonymous nature in accordance with the principles of the Declaration of Helsinki and the EU data protection regulations. Before taking part in the survey, participants had to provide informed consent digitally. The outpatients who had participated in study 1 were not included in study 2. The present investigation was part of a larger online survey. Findings from the same sample but related to other assessments and research questions have been reported elsewhere [44]. Participants did not receive any type of compensation or reimbursement.

#### 3.1.2. Instruments

The survey started with questions regarding demographic information, followed by the shopping-related dilemmas, a list of everyday moral dilemmas, and a battery of standardized questionnaires. For ethical reasons, the participants had the option to skip questions. To minimize the missing data due to unintended skipping, participants were informed about missing responses.

Symptoms of CBSD were assessed with the PBS [42,43] (see Section 2.1.2).

To investigate domain-specific shopping-related decision making, the 22 shopping-related dilemmas developed in study 1 (Table 1) were presented. Each dilemma closes with a question that had to be answered with “yes” or “no”, indicating either a more ego-oriented (coded 1) or a more norm-oriented (coded 0) decision alternative. For the purpose of model testing, sum scores of the 22 responses (including 5 reversely coded items, see Table 1) were built. Higher scores correspond to more ego-oriented shopping-related decision-making.

To explore everyday moral decision making, 20 close-to-everyday forced-choice moral dilemmas were used. These were already utilized and validated in other studies [41,45,46]. The dilemmas were kindly provided in the German and English languages by Dr. Katrin Starcke (SRH Berlin University of Applied Sciences). They represent dilemma situations that could potentially occur in everyday life and are less complex than the variations of the trolley or footbridge scenarios where the number of saved lives matters [33]. Each dilemma (answered with “yes” or “no”) offered a more egoistic or more altruistic alternative. The dilemmas are listed in the paper published by Starcke et al. [41]. In the following, two examples are presented. (1) “You have slightly scratched a car while parking. It is dark and nobody has seen you. Would you leave a message for the owner of the car? (no = egoistic answer, coded 1; yes = altruistic answer, coded 0), (2) “Your partner is suicidal and you feel uncomfortable in this relationship. Would you leave your partner?” (no = altruistic answer, coded 0; yes = egoistic answer, coded 1). For model testing, sum scores of the 20 responses were calculated with higher scores indicating more egoistic moral decisions.

Materialistic values endorsement was measured by means of the German translation [47] of the short Materialistic Values Scale (MVS) [20] that consists of 15 items rated on a scale ranging from 1 (=not true) to 5 (=completely true). In the model testing, items that directly refer to buying things were removed in order to discriminate between CBSD and materialism (i.e., item 6 “I’d be happier if I could afford to buy more things.”, item 14 “‘Buying things gives me a lot of pleasure.”, item 15 “‘It sometimes bothers me quite a bit that I can’t afford to buy all things I’d like.”). Higher MVS total scores (range 12 to 60) indicate higher levels of materialistic values endorsement (12-item MVS, α = 0.80 in the present sample).

#### 3.1.3. Participants

The survey included 274 internet users between 18 and 81 years (*M* = 34.88, *SD* = 13.76, *Mdn* = 29, *IQR* = 23). Most participants were young adults (18–24 years *n* = 79, 25–34 years *n* = 87, 35–44 years *n* = 32, 45–54 years *n* = 45, 55–64 years *n* = 25, and ≥65 years *n* = 6). Information regarding gender was available from 271 individuals. Of those, 198 were women (73.1%), 71 men (26.2%), and two persons (0.7%) defined themselves as diverse. Two hundred and seventy two participants provided information regarding their nationality (German, *n* = 260, 95.6%), 273 responded to the question about school years (≥11 school years, *n* = 241, 88.3%) and 266 reported their employment/occupational status (employed, *n* = 152, 57.1%; students, trainees or apprentices, *n* = 94, 35.3%). Responses to shopping-related dilemmas, close-to-everyday moral dilemmas, PBS and MVS questions were provided by all participants.

#### 3.1.4. Statistical Analyses

All analyses were carried out with SPSS version 26 (IBM Corp., Armonk, NY, USA). Bivariate one-tailed Pearson correlations were calculated for all variables, except for the relationship between age and PBS scores. Due to the skewed distribution of age, a one-tailed Spearman’s *r* was used. Fisher’s z values were used to test the difference between correlations.

The association of shopping-related decision making with materialism, CBSD symptoms and everyday moral decision-making (see Figure 1) was analyzed with a moderated hierarchical regression analysis with the means of ego-oriented shopping-related decisions as the dependent variable. Centralized means of the MVS, PBS and egoistic everyday moral decision making were entered in step 1. To test the possible moderating effects of egoistic everyday moral decision-making on the relationship between materialism (=predictor)/CBSD symptoms (=predictor) and ego-oriented shopping-related decision making (2-way interactions), the interactions “MVS/PBS × egoistic everyday moral decision making” were included in step 2. In addition, the 2-way interaction “MVS × PBS” was analyzed. Given that materialistic values endorsement was conceptualized as an antecedent of CBSD [22,23,29,30], materialism was considered as predictor and CBSD symptoms as moderator in the model. The interaction terms were calculated by using the centered values of the predictor and the moderator variables [48]. In step 3, a possible 3-way interaction was tested by entering the “MVS × PBS × everyday moral decision making” term in the model. Multicollinearity was evaluated with the variance inflation factors (VIF). Simple slope analyses were performed to illustrate the significant interaction effects. Predictors/moderators were grouped one standard deviation above and below the mean to explore high and low values [49]. For all variables, values of *p* < 0.05 were considered to be statistically significant.

### 3.2. Results

By applying the PBS-threshold, 34 participants (of those 73.5% women) were at-risk for CBSD, indicating an estimated point prevalence of 12.4% in the total sample. Younger age was weakly correlated with higher PBS scores (*r_s_* = 0.22, *p* < 0.01). Frequencies of being at-risk for CBSD across different age categories were: 18–24 years 19.0%, 25–34 years 9.2%, 35–44 years 6.3%, 45–54 years 15.6%, 55–64 years 8.0%, and ≥65 years 0%. Descriptive statistics and one-tailed zero-order correlations between main variables are displayed in Table 2. More ego-oriented shopping-related decisions correlated low to moderate with materialism and symptom severity of CBSD, and materialism was significantly correlated with CBSD symptoms. The link between ego-oriented shopping-related decisions and CBSD symptoms was stronger than the link between ego-oriented shopping-related decisions and materialism (*z* = 2.69, *p* = 0.004) or between CBSD symptoms and materialism (*z* = 2.51, *p* = 0.006). No significant correlations were found between everyday moral decisions and ego-oriented shopping-related decisions or CBSD symptoms. There was a significant, albeit weak, link between everyday moral decision-making and materialism.

**Figure 2:**
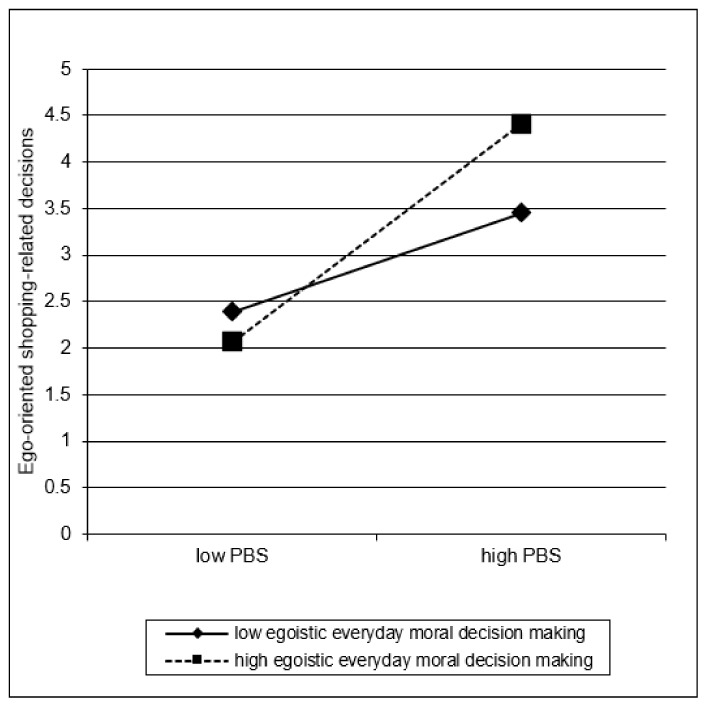
Results of the regression analysis indicating the interaction between compulsive buying-shopping disorder symptoms (Pathological Buying Screener, PBS) and everyday moral decision making on ego-oriented shopping-related decisions. Note that the values “low/high” represent the predicted ego-oriented shopping-related decisions, based on the regression coefficients, when a value of one standard deviation below the mean (=low) and a value of one standard deviation above the mean (=high) of the predictor (PBS) and moderator (everyday moral decisions) is used. More buying-shopping disorder symptoms (=higher PBS scores) are related to more ego-oriented shopping-related decisions, whereas an egoistic everyday moral-decision making style strengthens this connection.

**Figure 3:**
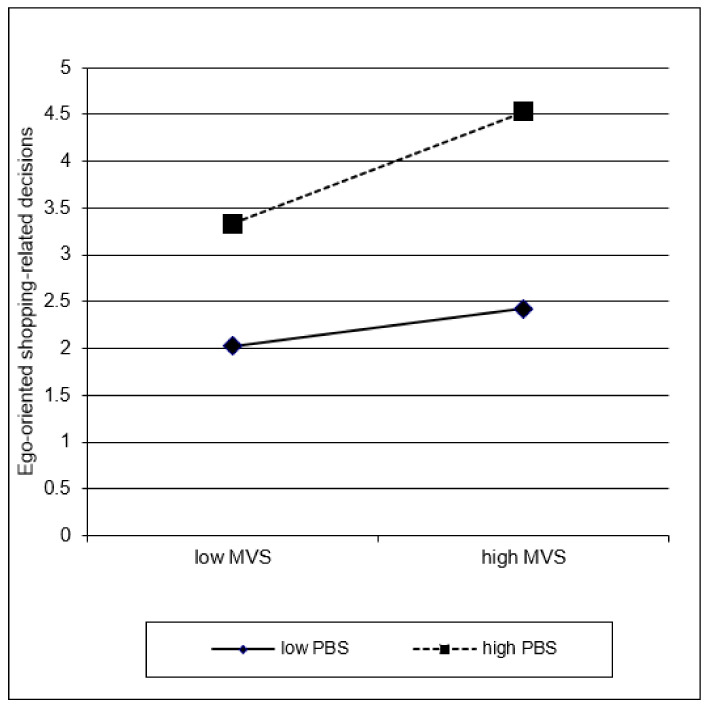
Results of the regression analysis indicating the interaction between materialistic values endorsement (MVS) and compulsive buying-shopping disorder symptoms (Pathological Buying Screener, PBS) on ego-oriented shopping-related decisions. Note that the values “low/high” represent the predicted ego-oriented shopping-related decisions, based on the regression coefficients, when a value of one standard deviation below the mean (=low) and a value of one standard deviation above the mean (=high) of the predictor (MVS) and moderator (PBS) is used. More materialistic values (=high MVS) are related to more ego-oriented shopping-related decisions, particularly in individuals with high symptom severity of buying-shopping disorder (=high PBS).

Table 3 shows the summary of the moderated regression analysis. In step 1, materialism (MVS) and CBSD (PBS) but not egoistic everyday decision making were significantly associated with more ego-oriented shopping-related decisions [*F* (3, 270) = 37.15, *p* < 0.001; *R^2^* = 0.29]. When adding the two-way interactions in step 2, the change in *R^2^* was significant [*F* (6, 267) = 21.34, *p* < 0.001; Δ*R^2^* = 0.03, *p* < 0.001]. Materialism (MVS) and symptom severity of CBSD (PBS) were still predictive for ego-oriented shopping-related decisions. In addition, “MVS × PBS” and “PBS × egoistic everyday moral DM” interactions showed a mild effect. Including the 3-way interaction “MVS × PBS × egoistic everyday moral decision making” in step 3 did not contribute to variance explanation (Δ*R^2^* < 0.01). The overall model explained 33% of the variance [*F* (7, 266) = 18.45, *p* < 0.001; *p* < 0.001]. The level of multicollinearity was low (all VIF ≤ 1.87). Figure 2 and Figure 3 illustrate the significant two-way interactions. Severe CBSD (=high PBS) was related to more ego-oriented shopping-related decisions, whereas this link was strengthened by high egoistic everyday moral decision making (Figure 2). Figure 3 indicates that high materialistic values endorsement (=high MVS) was related to more ego-oriented shopping-related decisions, particularly in individuals with high symptom severity of CBSD (=high PBS).

## 4. Discussion

The present work is one of the first investigations addressing ego-oriented shopping-related behaviors and their relationship with symptoms of CBSD and other potentially relevant variables. The findings supported hypothesis 1, that high materialistic values endorsement is associated with ego-oriented shopping-related decision-making. This result is in line with previous research that indicates that materialism is connected with ego-oriented shopping motivations, poor money management skills, more favorable attitude toward debt, or treating others in self-serving ways [19,21,29,31,50].

The results also supported hypothesis 2. More symptoms of CBSD were associated with more ego-oriented shopping-related decisions. The outcome suggests that a higher level of CBSD symptoms is accompanied by a higher level of ego-oriented shopping-related decisions. This is in agreement with case vignettes [10,11,15] and indicates that norm-breaking shopping-related behavior is a qualifier of CBSD severity. It is possible that a domain-specific, disadvantageous, shopping-related decision making style is especially significant in the later stage of the addiction process when individuals with CBSD run into high debts [8,51], hide the extent of over-spending from their relatives [52], and may have financial legal consequences [9,10,11,15]. In this stage, the excessive, dysfunctional shopping behavior has probably become seemingly automatic [12,13]. However, because of the cross-sectional study design and the lack of information about the duration of CBSD symptoms in participants of the online survey in study 2, we cannot derive causal interpretations. Therefore, these assumptions should be addressed in a longitudinal study.

Interestingly, more egoistic everyday moral decision-making was not directly related to ego-oriented shopping-related decisions, therefore not supporting hypothesis 3. This finding could be (mis)interpreted as if moral decision-making does not matter in shopping-related reasoning. However, an important outcome of the present study is that egoistic moral decision making moderated the effect of CBSD symptoms on shopping-related decisions, which partially supported hypothesis 4. While the symptom severity of CBSD is a crucial factor affecting ego-oriented shopping-related decisions, egoistic everyday moral decision making makes that connection stronger (Figure 2). This complex interplay should be considered when assessing and treating CBSD. Cognitive-behavioral therapy has been shown to be effective in the treatment of CBSD [53,54,55,56]. Individuals with CBSD who admit to a high level of socially unacceptable shopping-related activities (e.g., fraud, embezzlement, shoplifting, income-generating crime) should be offered additional modules that target their intuitive general moral beliefs and attitudes. It is worth inducing those patients to be more reflective in terms of moral judgment, features of antisocial thinking, recklessness and treating other people in self-serving ways. One option would be to implement more mentalization-based approaches in the treatment of CBSD.

Materialism was significantly, albeit moderately, correlated with CBSD symptoms, which replicated past findings [21,22,23,24,25,26,27,28]. Ego-oriented shopping-related decisions were related to both materialism and CBSD, whereas the bivariate correlations and main effects of the regression model indicated the strongest association between ego-oriented shopping-related decisions and CBSD symptoms. On the one hand, it may be argued that the close link is not surprising given that the scenarios of ego-oriented shopping-related decisions were developed based on interviews with patients with CBSD. On the other hand, it seems plausible that norm-breaking shopping-related decisions are more directly connected with CBSD than with materialism. Along the same lines, the simple slope analysis (Figure 3) showed that the link between materialism and ego-oriented shopping-related decisions was particularly strong among individuals with high symptom severity of CBSD.

The development of shopping-related dilemmas by using a qualitative approach in study 1 is an advantage of the present investigation because the list of conflict situations used in study 2 is probably close to the reality of persons suffering from CBSD and has good ecological validity. The scenarios may also serve as a resource for the development of a compact, standardized list of CBSD-related dilemmas in future research.

### Limitations

Notwithstanding the strengths of the study, there are also shortcomings. The shopping-related dilemmas developed in study 1 were not validated before using them in study 2. By analogy with moral dilemmas [40,41,57], they should be examined with regard to arousal, valence, item difficulty, reliability, ambiguity, lengths, and expression style in further studies. It is also important to emphasize that all assumptions from study 2 can only be made for the present web-based convenience sample. Furthermore, the relatively high amount of participants at-risk for CBSD in study 2 (12.4%) indicates a selection bias that might be due to the invitation to take part in an online survey concerning “buying-related decisions”. The high number of female participants (73.1%) may constitute another reason for the relatively high proportion of participants at-risk for CBSD. Population-based studies indicate that women are more likely to suffer from CBSD than men [42,58,59,60]. It is unclear how this selection bias influenced the results. While studies concerning gender differences in moral reasoning reported mixed results, it appears that women are more likely to adhere to rules than men [61,62,63,64]. Hence, one may assume that the interaction of egoistic moral decision making and materialism/CBSD on ego-oriented shopping-related decisions would have been stronger in a male sample. However, this interpretation remains speculative. Finally, the study did not consider emotional states, cognitive mechanisms, theft behaviors and comorbidity with hoarding disorder or kleptomania that may also influence shopping-related and moral decision-making.

## 5. Conclusions

The present findings indicate that more symptoms of CBSD are linked to ego-oriented shopping-related decisions and that a more egoistic everyday moral decision-making style strengthens this relationship. The higher the level of egoistic moral decision making, the stronger the association between CBSD and norm-breaking shopping behaviors. Some clinical implications can be derived from the findings. The assessment of CBSD should consider norm-breaking shopping-related behaviors as a qualifier of the severity. Clinicians should inquire about altered moral decision making that may contribute to the negative consequences of CBSD. In this sense, preventive and intervention efforts should not avoid addressing malfeasance, norm violations, crime and antisocial features related to CBSD.

To prove the generalization of the present findings, their replicability should be examined using a clinical sample with patients suffering from CBSD. Given the expansion of the e-commerce marketplace, research should address the question whether shopping-related norm-breaking behaviors differ between excessive in-person and online shopping. Also, possible gender differences in norm-breaking shopping-related behaviors should be investigated. Moreover, future longitudinal studies or those including participants in early and later stages of CBSD should investigate the trajectories of ego-oriented shopping-related decisions over time.

## Figures and Tables

**Table 1:** List of exemplary scenarios representing a shopping-related decisional conflict situation.

Answer		Dilemma
Norm-related answer = no Ego-related answer = yes	1	It is the middle of the month and the money in your bank account to pay all necessary expenses will presumably just last until the end of the month. Only then will you receive the next payment (e.g., salary, student loan, pension, unemployment benefits, support from relatives, etc.). You would like to treat yourself and buy something nice. However, by buying something you would probably overdraw your bank account. Do you buy something nice for yourself?
	2	You would like to order an item, but you currently do not have enough money. Would you order the item in the name of another person instead?
	3	You come across an item you would really like to have. Only yesterday you were paid money with which you can finally pay back the amount of money you are owing to a family member. Would you use the money to buy the item instead of paying the money back you are owing?
	4	You are visiting a shop and a sales assistant offers detailed advice. You originally had no intention of buying anything, but feel obliged to do so due to the service you have received. Do you buy something?
	5	You would like to buy something online. However, you still have outstanding invoices at this online retailer which prevents you to order from there. Do you place your order under another person’s name?
	6	You are currently short on cash and have recently returned an item you had ordered. So far, the refund has not been credited to your account. You see another item you would like to have. Do you buy that item?
	7	Only yesterday you were paid money to settle an outstanding invoice for medication. Now you discover an article you would like to have and consider whether you should buy the article with that money. Do you buy the item?
	8	You know that some repairs are due in your household soon, e.g., for your washing machine or your car, and you have put money aside. You see an item you would like to have. Do you buy the item?
	9	You have a guilty conscience because you have spent too much money lately. Do you still go shopping?
	10	You are annoyed with a person who is close to you. You know that he or she dislikes it when you spend money on things that are not absolutely necessary. Do you now buy something nice for yourself?
	11	You have discovered an item you would like to have which is currently on sale. You do not want to miss out the opportunity, but you do not have the money for it in the foreseeable future. Do you ask a friend or family member for the money to buy the item?
	12	You have bought an item that you would like to use. However, your relatives or people from your circle of friends have often criticised your buying behaviour, which has already led to arguments. Do you hide the item from the others?
	13	You divide your money into a weekly allowance, have set a weekly shopping list and a weekly budget. You see an item that you did not plan to purchase, but still would like to have. Do you buy the item with money from your weekly budget?
	14	You are very short on cash at the moment. You have had a bad day and you know that a stroll through the shops usually improves your mood. However, you also know that then the temptation to actually buy something is quite high. Do you go on a shopping spree?
	15	You often order goods and have them delivered to your house. You share your house with your relatives who wonder about all of those packages. You do not know how to react to the queries and are ashamed of your buying behaviour. Do you tell your relatives that you did not order the goods for yourself but for another person who has already given you the money for them?
	16	You have had unpaid accounts in recent times because you have made too many purchases. In order to settle these invoices you would like to ask a family member, who helped you out financially previously, to lend you the funds. However you are worried this person will not give you the money, once he/she finds out what it is for. Will you ask this person for the money under the pretext it is for something else?
	17	You are currently in a difficult financial situation due to private and family related problems. You would really like to treat yourself by buying something nice. You see an item on display, which you really like and would like to have. Currently you do not have the funds. Will you take the item, without paying for it?
Norm-related answer = yes Ego-related answer = no	18	You are looking at an item that you would like to buy because you like it a lot. At the same time, you are aware that it would be good to save the money for an activity, e.g., going to the theatre, the cinema or the gym. Do you save the money for an activity?
	19	You like to give big and expensive gifts. You have incurred debts through your increased spending. You are invited to a birthday party. Do you spend less on the birthday present this time than usual?
	20	You enjoy shopping for your family and yourself. You pay with the money from the joint account with your partner. Due to your spending habits you have incurred debts which your partner does not know. Do you tell him or her about the debts?
	21	You have ordered some items in the name of a family member, but cannot pay the bill. You fear that this family member might soon receive a reminder monition. Do you tell him or her that you have ordered the items on the internet in his or her name?
	22	You have ordered items online which you have received now. You do not really like them. Are you going to return the items?

^1^*Tables Note.* Dilemmas were originally created in the German language. For the purpose of presentation in the manuscript, they were translated into English language by three independent persons. Consent as to wording was reached by discussion. Finally, the English translations of the dilemmas were reviewed by an English native speaker for colloquial speech and concordance with the original German dilemmas.

**Table 2:** Means (*M*), standard errors (*SE*), confidence intervals *(CI)* and Pearson correlation coefficients *r* (*n* = 274).

	*M (SE)* *95% CI*	Material Values Scale	Pathological Buying Screener	Egoistic Everyday Moral Decisions
Ego-oriented shopping-related decisions	3.19 (0.14) [2.92, 3.46]	0.35 ***	0.51 ***	0.09
Material Values Scale	25.42 (0.43) [24.58, 26.27]	-	0.36 ***	0.18 **
Pathological Buying Screener	22.13 (0.42) [21.31, 22.96]	-	-	0.05
Egoistic everyday moral decisions	9.66 (0.15) [9.36, 9.97]	-	-	-

*** *p* < 0.001, ** *p* < 0.01.

**Table 3:** Summary of the regression analysis investigating the prediction of ego-oriented shopping-related decisions (dependent variable) by materialistic values endorsement (MVS), symptoms of compulsive buying-shopping disorder (PBS) and egoistic everyday moral decisions.

	B	SE	95% CI	β	*t*	*p*
**Predictor/moderator variables**						
MVS	0.06	0.02	[0.02, 0.09]	0.18	3.16	0.002
PBS	0.12	0.02	[0.08, 0.16]	0.38	6.13	<0.001
Egoistic everyday moral DM	0.06	0.05	[−0.03, 0.16]	0.07	1.28	0.200
**2-way interactions**						
MVS × PBS	0.00	0.00	[0.00, 0.01]	0.12	2.09	0.038
MVS × egoistic everyday moral DM	0.01	0.01	[−0.01, 0.02]	0.05	0.99	0.325
PBS × egoistic everyday moral DM	0.02	0.01	[0.00, 0.03]	0.15	2.14	0.033
**3-way interactions**						
MVS × PBS × egoistic everyday moral DM	0.00	0.00	[−0.00, 0.00]	−0.07	−1.03	0.302

CI = Confidence Interval, MVS = Material Values Scale, PBS = Pathological Buying Screener, DM = decision making.

## Data Availability

The data of study 1 will be available through a direct request to the authors, who will evaluate the type of information requested with the Ethics Committee at the Hannover Medical School. Due to the clinical nature of the sample analyzed and the fact that the patients signed a consent form for their data to be stored and kept by the two recruiting hospital centers, it is not possible to transfer them to an open data repository. The data presented in study 2 are openly available at https://doi.org/10.26068/mhhrpm/20220404-001 (accessed on 10 February 2022).
